# Misconceptions about COVID-19 vaccine among adults in Saudi Arabia and their associated factors: A cross-sectional study conducted in 2021

**DOI:** 10.12688/f1000research.110270.2

**Published:** 2023-12-27

**Authors:** Fatma I. Albeladi, Eman A. Kubbara, Marwan A. Bakarman, Turki Al Amri, Rasha Eid, Najla Alyazidi, Ameera Alkhamesi, Atheer Alasslany

**Affiliations:** 1 Department of Nephrology, Faculty of Medicine, King Abdul-Aziz University, Rabigh, Makkah, 25724, Saudi Arabia; 2 Department of Clinical Biochemistry, Faculty of Medicine, King Abdulaziz University, Rabigh, Makkah, 25724, Saudi Arabia; 3 Department of Clinical Biochemistry and molecular biology, Faculty of Medicine, Al-Neelain University, 52nd St, Khartoum, 11121, Sudan; 4 Department of Family Medicine, Faculty of Medicine, King Abdul-Aziz University, Rabigh, Makkah, 25724, Saudi Arabia

**Keywords:** COVID-19; vaccination; vaccine hesitancy; acceptance; misconceptions; rumors, misinformation; predictors.

## Abstract

**Background:**

It is of utmost importance for the elements that influence public compliance with vaccination against COVID-19 to be assessed, including misconceptions, rumors, and conspiracy theories. Hence, in this study, we aimed to estimate the distribution of the most common misconceptions regarding COVID-19 vaccines and their predictors in Saudi Arabia.

**Methods:**

We distributed an online questionnaire to participants aged 18 years or older. The survey included two sections. The first section comprised questions related to participants’ demographic characteristics, level of education, and their sources of information about COVID-19. The second section assessed participants’ perceptions regarding 11 of the most common misconceptions regarding COVID-19 vaccines, rated using a 5-point Likert scale. Using ordinal logistic regression, we conducted an evaluation of the relationships among different predictors including age, sex, educational level, and sources of information, as well as acceptance of misconceptions about vaccination.

**Results:**

The most widely accepted misconception was that the COVID-19 vaccine had severe side effects, with 34.8% of participants believing this misinformation. Factors that were significantly associated with acceptance or non- acceptance of misconceptions were: 1) sex, with female respondents in this survey accepting rumors significantly more often than male respondents (p<0.001); 2) educational level, especially secondary school, was associated with a significantly lower acceptance of misconceptions (p=0.001). In total, 60.5% of participants used social media as their primary source of information, which was also a significant positive predictor of acceptance of misconceptions (p=0.034).

**Conclusion:**

It is of critical importance to increase assurance regarding the safety of COVID-19 vaccines, the issue most likely to involve misconceptions, and to address the elements that affect belief in rumors among the population.

## Introduction

COVID-19 has transformed the world since its emergence in December 2019, leading to a very large number of infected cases and deaths in 213 countries, as of 23 December 2020. ^1^ The earliest cases likely occurred in October or November 2019, according to molecular clock inference studies. ^2^ Vaccination has led to the eradication or reduction of many infectious diseases, including smallpox, rubella, polio, and measles. ^3^ Thus, to combat SARS-CoV-2, the virus that causes COVID-19, and prevent its continued spread globally, numerous vaccines have been developed. However, acceptance of these vaccines has faced challenges in the form of hesitancy, non-acceptance, and belief in conspiracy theories. Vaccine skepticism has been highlighted as a worldwide health threat. ^4^

The first COVID-19 vaccine human trial started in March 2020. Several other trials began shortly after that, and by September of the same year, eight vaccines had either progressed to phase ІІІ clinical trials or had been approved for use. However, many people remain skeptical about the vaccines. A study performed in several countries of Europe revealed that 7.8% of respondents are unwilling to be vaccinated. ^5^ Several vaccine candidates have been developed using different approaches, such as viral vectors, RNA, and single proteins. Some vaccines have been approved for use in multiple countries, such as the in United States and Saudi Arabia. ^6^ In December 2020, the Ministry of Health developed a mobile application in Saudi Arabia, known as Sehaty, and vaccination centers were established in various regions across the country. ^7^ Vaccines that were initially approved for use in susceptible groups later became accessible to all populations. ^8^ Subsequently, the COVAX initiative for global access to COVID-19 vaccines was initiated by the World Health Organization and numerous countries to speed the production of vaccines against the disease and provide unbiased access to vaccines internationally. ^9^ The effective final price of a new vaccine may vary depending on various factors that the manufacturers may consider when it comes to pricing, which is also an essential factor when considering vaccine hesitancy. ^10^ Vaccines should have at least 50% efficacy against the virus to effectively fight the spread of infection. ^11^ To reach sufficient immunity, a large proportion of the population should be vaccinated, and this mostly depends on public acceptance or rejection of vaccination. ^11^

Many factors contribute to compliance with vaccination against COVID-19, including male sex, older age, and fear of infection. Also, being married can boost the likelihood of receiving the vaccine. A recent study revealed that approximately 68% of Saudi respondents planned to receive the COVID-19 vaccine and 7% were hesitant. The study also found that compliance with vaccination was higher among well-educated respondents, non-Saudis, and government employees. ^12^ A study done in 2020 revealed that a high percentage of individuals reported that they would receive the COVID-19 vaccine if it became accessible. ^13^ Vaccine hesitancy has been demonstrated even among health care workers, especially nurses. ^14^ However, a recent study in Vietnam revealed contradictory results, with a high acceptance rate (76.1%) among health care workers. ^15^

In wealthy countries like the United States, so called anti-vaxxers are widely opposed to vaccination owing to a belief in the correlation of vaccines with autism and other adverse effects. The anti-vaccination movement has led to measles outbreaks in some parts of the country, highlighting the danger of misconceptions and false beliefs in preventive health care delivery. ^16^ Other essential predictors of vaccine hesitancy are complacency owing to underestimation of the risk of a contracting a disease. Confidence in vaccination is related to vaccine safety, efficacy, and trust in the health system, as well as convenience, which involves the affordability and availability of vaccines. ^17^ A recent survey in Bangladesh found significant rates of refusal of the COVID-19 vaccine in groups such as older people, day laborers, and those who were skeptical of the country’s health policy. ^18^

In countries like the United States, the most vaccine-hesitant individuals are those with lower income levels and no health insurance. These individuals are also more likely to be women and Black. ^19^ Various factors have been identified that explain why some people avoid being vaccinated, including concerns about safety and effectiveness. ^20^ It has been revealed that a small percentage of the population has anti-vaccination beliefs, and a larger portion can be considered vaccine-skeptical. ^21^

Vaccine hesitancy or refusal could be partly related to the dissemination of incorrect information on social media; 30%–60% of information on social media platforms tends to be anti-vaccination, and over 50% of the information on websites that discuss vaccination is false or inaccurate. ^22^

In this study, we aimed to assess the most widely accepted misconceptions among adults in Saudi Arabia regarding vaccination against COVID-19 and to investigate the predictors that can increase or decrease acceptance or refusal of vaccination among the general public.

## Methods

### Ethics

This study was approved by the Unit of Biomedical Ethics Research Committee at King Abdulaziz University, Saudi Arabia (Reference No. 254-21). Participation in the survey was voluntary, and informed consent was electronically provided by all respondents to the online survey. All collected data was treated with confidentiality.

We administered an online cross-sectional survey in Saudi Arabia between 21 ^st^ April and 28th December in 2021. All adult male or female individuals (citizens and residents) aged 18 years or older at the time of the survey and living in the Kingdom of Saudi Arabia during the study period were eligible to participate. Data were collected using a Google Forms-based questionnaire, and invitations were distributed via social media or e-mail.

The sample size was calculated using the formula: (n=Z2*p (1-p)/m2, where: n=1.962*0.5(1-0.5)/0.052=384.1), ^23^ the minimum number of participants that should be accepted in the survey was 384, the number was increased to 1131 respondents.

The questionnaire began with information about the study purpose and an explanation of the confidentiality of personal information. Informed consent was obtained from all respondents before they could proceed with the questionnaire; participants were informed that they could withdraw at any time.

We conducted a pilot study among 10% of the study population (n=113) to test the applicability of the questionnaire and accessibility for the study sample. This step helped in determination of the required organization and administration procedures for the study and revealed the difficulties in design and language that could arise in the survey. The questions were in Arabic, which was the official language of all participants. The survey was translated into English and then back-translated into Arabic to ensure that the meaning was not changed. A copy of the survey can be found in *Extended data.*

This study was conducted to assess the main misconceptions among the public and their predictors regarding vaccination against COVID-19. Respondents’ perceptions toward different misconceptions were evaluated using a 5-point Likert scale, where 5 indicated “strongly agree” and 1 indicated “strongly disagree.”

### Survey items

The survey consists of two parts. The first section included questions on participants’ sociodemographic characteristics, including sex, age, profession, education level, and their sources of information regarding COVID-19. The second section addressed 11 misconceptions derived from the literature, the community, social media, and websites. ^24^^–^^26^ Respondents were asked to use a 4-point categorized scale to report the extent to which they agreed or disagreed with each of the 11 points (1=strongly disagree and 4=strongly disagree). The 11 misconceptions discussed were as follows. 1) I do not believe in the safety of the COVID-19 vaccines. 2) My genetic material will be affected by COVID-19 vaccines. 3) A device will be implanted in my body via the COVID-19 vaccines. 4) The COVID-19 vaccines have serious side effects, such as causing severe allergy. 5) Fertility is decreased in women who received a COVID-19 vaccine. 6) I have been infected with COVID-19, so vaccination is unnecessary. 7) Once I am vaccinated, I don’t have to wear a face mask. 8) A COVID **-**19 vaccine will change my test results to positive. 9) I am unlikely to have complications from COVID-19, so it is not necessary for me to be vaccinated. 10) You can get COVID-19 from the vaccine. 11) If I am vaccinated, I am more likely to get another disease.

### Statistical analysis

IBM SPSS software 28.0 was used for the analysis (IBM Corp., Armonk, NY, USA). We calculated the number and proportion of each predictor (age, sex, education level, and sources of information). The relationship between different predictors and acceptance of misconceptions was estimated using ordinal logistic regression. Log odds were estimated at 95% confidence intervals (CIs), and *p*-values less than 0.05 were considered significant. Visualization of the data was carried out using Tableau 2021.4.

## Results

Table 1 shows participants’ sociodemographic characteristics. A total of 1131 respondents were included, with 60.5% women and 39.5% men. Most respondents (37.4%) were in the age group 18–30 years, followed by the age groups 31–40 years (34.7%), 41–50 years (19.6%), and 51–60 years (7.2%); only 1.1% of participants were in age group 61–70 years. In total, 50.1% of respondents were employed, followed by students (25.7%) and homemakers (14.3%). Most participants had a university-level education (68.7%); 20.4% completed secondary school, 9.0 % of respondents had an education level above university, and 1.9% had education levels below secondary school.

**Table 1.** Distribution of participants according to their demographic characteristics.

Demographic characteristics		
	Number	Percent
**Gender**		
**Female**	684	60.5%
**Male**	447	39.5%
**Age group**		
**18–30**	423	37.4%
**31–40**	393	34.7%
**41–50**	222	19.6%
**51–60**	81	7.2%
**61–70**	12	1.1%
**Profession**		
**Employee**	567	50.1%
**Student**	291	25.7%
**Housewife**	162	14.3%
**Unemployed**	75	6.6%
**Pensioner**	27	2.4%
**Other**	9	0.8%
**Educational level**		
**University**	777	68.7%
**Secondary**	231	20.4%
**Above University**	102	9%
**Below Secondary**	21	1.9

Table 2 shows that 60.5% of participants obtained their information regarding COVID-19 from social media platforms like WhatsApp, Facebook, and Twitter. This was followed by traditional media, such as newspapers and television. In total, 21.0% and 9.0% of respondents relied on websites found using the Google search engine, 4% relied on scientific journals, and 0.8% obtained their information from YouTube.

**Table 2.** Distribution of participants depending on their sources of information regarding Covid-19.

	Number	Percent
**Social media such as Facebook, WhatsApp, and Twitter**	684	60.5%
**Traditional media such as Press and television**	237	21.0%
**Google search**	102	9.0%
**Scientific journals**	45	4.0 %
**YouTube**	9	0.8%
**Other**	54	4.8%

**Figure 1.**
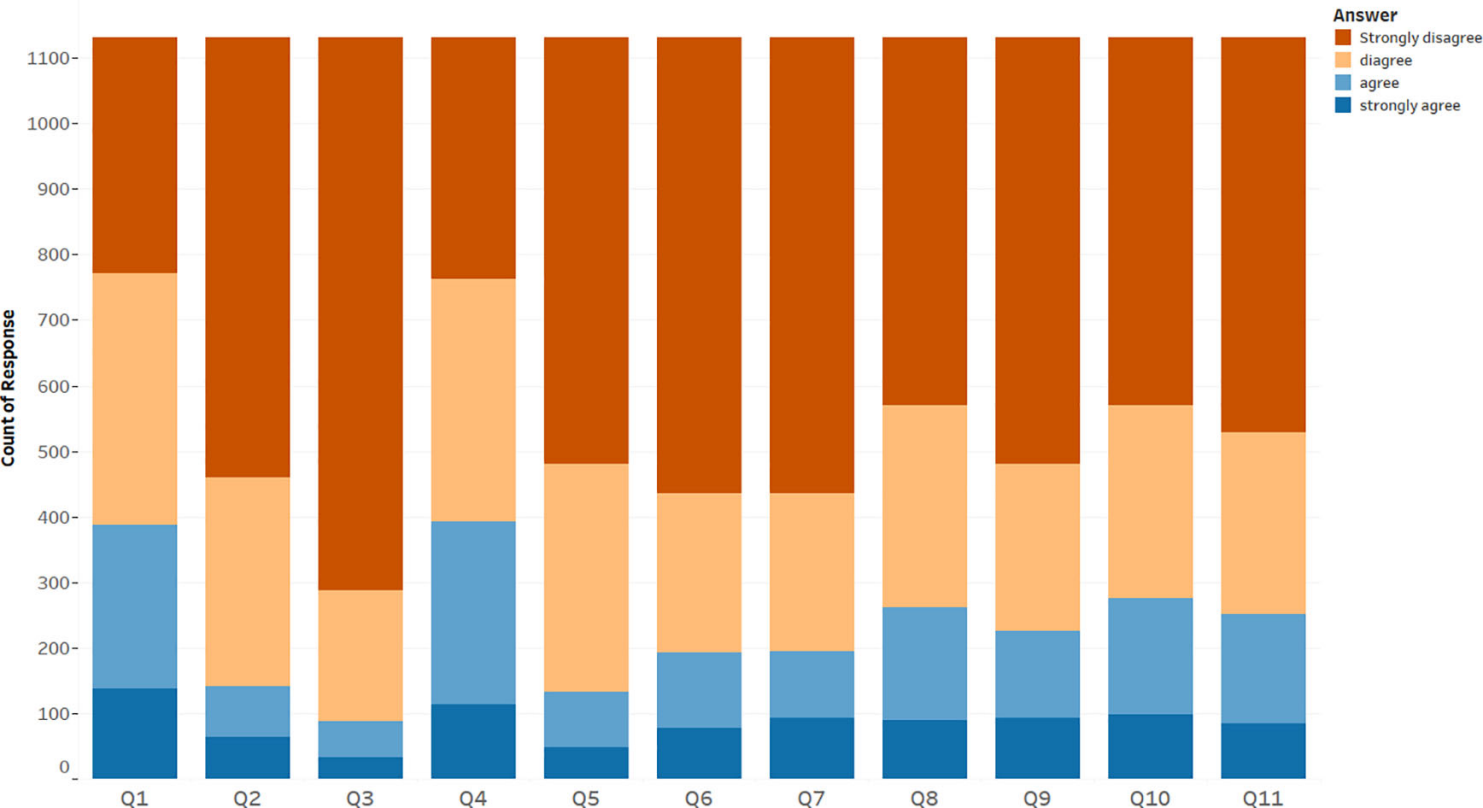
Distribution of participants regarding their acceptance and refusal of misconceptions.

Table 3 and Figure 1 show that the most widely accepted misconception among participants was that the COVID-19 vaccines have serious effects like causing allergy, with 34.8% of respondents either agreeing or strongly agreeing with this false information. This was followed by not believing in the safety of COVID-19 vaccines, accepted by 34.2% of participants; and the belief that COVID-19 can be contracted from the vaccine, with 24.4% of respondents either agreeing or strongly agreeing with this misconception; 23.1% of participants accepted the misinformation that COVID-19 vaccines change lab test results to positive, and 22.3% believed that they would develop another disease if they were vaccinated. Furthermore, 19.9% of respondents believed that they were unlikely to have COVID-19 complications so it was not necessary to be vaccinated; another 17.2% believed that once vaccinated, use of a face mask was unnecessary; 17% accepted the rumor that previous infection with COVID-19 meant that vaccination was unnecessary; 12.5% of respondents agreed or strongly agreed with the misconception that their genetic material can be affected by COVID-19 vaccines; and 11.6% believed that COVID-19 vaccines cause infertility among women. Finally, 7.7% of respondents either agreed or strongly agreed with the misinformation that COVID-19 vaccines contain a tracking device.

**Table 3.** The distribution of misconceptions showing the number and percentage of participants for each scale.

Misconceptions	Strongly disagree number (%)	Disagree number (%)	Agree number (%)	Strongly agree number (%)
**1. I do not believe in the safety of Covid-19 vaccine**	360 (31.8%)	384 (34.0%)	249 (22.0%)	138 (12.2%)
**2. My genetic material will be affected by COVID-19 Vaccine**	672 (59.4%)	318 (28.1%)	78 (6.9%)	63 (5.6%)
**3. A device will be implanted in my body using Covid-19 vaccine**	843 (74.5%)	201 (17.8%)	54 (4.8%)	33 (2.9%)
**4. The COVID-19 vaccine has serious side effects such as severe allergy**	369 (32.6%)	369 (32.6%)	279 (24.7%)	114 (10.1%)
**5. Fertility in women is decreased by Covid-19 vaccine**	651 (57.6%)	348 (30.8%)	84 (7.4%)	48 (4.2%)
**6. I got infected before with covid-19 so vaccination is unnecessary**	696 (61.5%)	243 (21.5%)	114 (10.1%)	78 (6.9%)
**7. Once I get vaccinated, I don’t have to put a facemask**	696 (61.5%)	240 (21.2%)	102 (9.0%)	93 (8.2%)
**8. Covid-19 vaccine will change my lab test result to positive**	561 (49.6%)	309 (27.3%)	171 (15.1%)	90 (8.0%)
**9. Covid-19 complications are not likely to happen to me, so it is not necessary to be vaccinated**	651 (57.6%)	255 (22.5%)	132 (11.7%)	93 (8.2%)
**10. You can get COVID-19 from the vaccine**	561 (49.6%)	294 (26.0%)	177 (15.6%)	99 (8.8 %)
**11. If I get vaccinated I am more likely to get another disease**	603 (53.3%)	276 (24.4%)	168 (14.9%)	84 (7.4%)

The most common source of information among respondents was social media, with 70.5% of male respondents and 53.9% of female respondents obtaining their information about COVID-19 from this source. The age group that most commonly used social media to obtain information regarding COVID-19 was 18–30 years (71.6%). Students also commonly used social media (70.1%) to find relevant information. Regarding educational level, social media was used most by respondents with a university degree (65.6%).

Table 4 shows the results of ordinal logistic regression, conducted to analyze the relationship among predictors including sex, age, profession, educational level, and acceptance of misconceptions. Sex was a significant positive predictor of acceptance of misconceptions ( *p*<0.001). The log odds of accepting misconceptions were 0.368 points higher, on average, for female than male respondents. Age was not a significant predictor of accepting misconceptions. However, the age group 18–30 years generally had the lowest rates of accepting misconceptions compared with other groups, although the difference was not significant.

**Table 4.** Ordinal logistic regression analysis of predictors in relation to vaccine misconceptions. (M=Male, F=Female).

Predictor	Odds (95%CI)	*p*-Value
Gender		
Female	0.368 (0.181–0.576)	<0.001
Age (M+F)		
18–30	-0.138 (-1.134–0.857)	0.785
31–40	0.217 (-0.779–1.214)	0.889
41–50	-0.018 (-0.990–1.028)	0.972
51–60	0.159 (-0.893–1.211)	0.767
Profession (M+F)		
Employee	-0.118 (-0.536–0.300)	0.580
Housewife	-0.049 (-0.524–0.426)	0.840
Other	1.156 (-0.044–2.357)	0.059
Pensioner	-0.591 (-1.355–0.174)	0.130
Student	-0.425 (-0.866–0.016)	0.059
Level of education (M+F)		
Below secondary school	0.329 (-0.423–1.081)	0.391
Secondary school	-0.420 (-0.676–0.164)	0.001
Above University	-0.100 (-0.459–0.258)	0.583
Sources of information (M+F)		
Google search	1.529 (0.329–2.730)	0.013
Other	2.313 (1.059–3.557)	<0.001
Scientific journals	0.729 (-0.528–1.997)	0.256
Social media such as Facebook, WhatsApp and Twitter	1.252 (0.093–2.411)	0.034
Traditional media such as Press and television	1.246 (0.074–2.418)	0.037

Regarding the profession of respondents, being a pensioner was associated with a decrease (−0.581) in the log odds of accepting misconceptions in comparison with other professions, although the association was not significant ( *p*=0.130). A secondary school education level was a significant negative predictor of acceptance of misconceptions ( *p*=0.001). The log odds of accepting misconceptions was lower (−0.420), on average, for secondary or high school educational levels. Furthermore, having an education above university level was associated with decreased log odds of acceptance of misconceptions, although the association was not significant. An education level below secondary school was a positive non-significant predictor of acceptance of misconceptions. Finally, using Google, traditional media like newspapers or television, and social media to obtain information about COVID-19 were all significant positive predictors of acceptance of misconceptions ( *p*=0.013, *p*=0.037, *p*=0.034, respectively).

## Discussion

Vaccination against COVID-19 is of paramount importance as new mutations of SARS-CoV-2 continue to emerge, which can increase the spread and severity of the disease. ^27^^,^^28^ Vaccination against COVID-19 faces many challenges and obstacles owing to a lack of acceptance or reluctance to receive the vaccine among the general public for reasons related to doubts about safety, efficacy, the health care system, or distrust of policymakers. ^29^ A global survey conducted in 19 countries involving more than 13,000 participants exploring compliance with COVID-19 vaccination showed differing proportions of acceptance, with a high rate (88.6%) in China. In contrast, 59%–75% was found in Western countries and rates as low as 54.8% were identified in Russia. ^4^ Saudi Arabia has a higher vaccination acceptance rate (64.7%), similar to many Western countries. ^30^

Despite the relatively high rates of compliance with COVID-19 vaccination in some countries, there is still a proportion of the population with vaccination hesitancy, which could be partly explained by the dissemination of some misconceptions and rumors that are likely to affect vaccine acceptance among the general public. In our study, misconceptions and rumors about the COVID-19 vaccine, which were distributed on social media or websites, were collected and scrutinized to better understand the factors that might affect their spread and acceptance.

We investigated 11 misconceptions, including the presence of a tracking device in vaccines. Only 7.7% of respondents either agreed (n=54, 4.8%) or strongly agreed (n=33, 2.9%) with this rumor. This percentage is small compared with those in other surveys conducted in many countries throughout the Middle East; in one survey, 27.7% of participants reported that they believed that the vaccines contain microchips that can be used to control people. ^31^

Another misconception was that COVID-19 vaccines can cause infertility in women. In our survey, 11.6% of respondents either agreed (n=84, 7.4%) or strongly agreed (n=48, 4.2%) with this misinformation. This percentage is lower than results of the above survey in which 23.4% of respondents reported that they believed COVID-19 vaccines can lead to sterility and the inability to conceive. ^31^ The smaller percentages of the abovementioned misconceptions found in our survey can be attributed to the considerable efforts made by Saudi health authorities to increase awareness about COVID-19 vaccination among citizens and residents.

The notion that COVID-19 vaccines have serious side effects like severe allergy was accepted by 34.8% of respondents who either agreed (n=297, 24.7%) or strongly agreed (n=114, 10.1%) with this rumor. This proportion is relatively high compared with other misconceptions examined in this study. One of the most likely reasons for acceptance of this misconception may be false news reports regarding the death of one participant in the COVID-19 Oxford vaccine clinical trial owing to disease complications and other false reports regarding complications caused by the vaccine. ^32^

It is worth noting that Covid-19 vaccine has been reported to cause adverse side effects in some individuals such as injection site pain, drowsiness, fatiguability and muscle pain, but severe side effects were rare. ^33^

Elhadi and colleagues reported that nearly one-third of study participants had concerns about serious complications owing to the vaccine. ^34^ Another survey conducted in countries throughout Latin America and the Caribbean confirmed these results, with a high percentage of the population feeling anxious regarding the vaccine’s adverse effects, especially in Venezuela (92.7%). ^35^ It is essential to consider the central part played by the media in disseminating such false information. As an example, the number of YouTube videos focused on the adverse effects of COVID-19 vaccines increased threefold from July to December 2020, with cumulative views increasing from 11.7% to 27.2%. ^36^ This was also proven in our survey, where we found that different sources of information, including Google searches and use of traditional media like newspapers or television as well as social media, were positive predictors of acceptance of misconceptions.

Doubts regarding the safety of COVID-19 vaccines were reported by 34.2% of respondents who agreed (n=249, 22.0%) or strongly agreed (n=138, 12%) shared these misconceptions. However, 65.8% of participants disagreed with this misconception, compared with the results of a survey conducted in Jordan where 56.8% of workers in medical fields believed that the vaccines are safe and 31.9% of non-medical workers shared this belief. ^37^

In the abovementioned survey, Abdelkarim Aloweidi and colleagues reported that 8.7% of medical workers and 17.3% of non-medical workers believed the rumor that COVID-19 vaccines can affect genetic material. These results are roughly similar to ours, where 12.5% of respondents either agreed (n=78, 6.9%) or strongly agreed (n=63, 5.6%) with this misconception. The lower proportion among those in the medical field compared with other disciplines is owing to greater knowledge about the vaccines’ mechanisms of action. Another misconception reported in the same survey was that there is an increased risk of contracting another disease after vaccination against COVID-19. The survey results showed that 7.7% of respondents in the medical field and 10.6% of non-medical workers believed this theory. In comparison, we found that 22.3% of respondents agreed (n=168, 14.9%) or completely agreed (n=84, 7.4%) with this misconception, which is higher than in that previous study.

The belief that it is not necessary to receive a COVID-19 vaccine because one feels they are unlikely to develop any complications from the disease is also common in some populations. In our sample, 19.9% of respondents either agreed (n=132, 11.7%) or strongly agreed (n=93, 8.2%) with this misconception. This is supported by the results of a survey among nurses in Hong Kong, where 27.9% of respondents agreed that vaccination was unnecessary for this reason. ^38^ Comparable results were also found in China, with 29.2% of participants unwilling to be vaccinated because they thought they were healthy and had a low probability of complications from COVID-19 infection. ^39^

Another incorrect theory explored in our survey was that previous infection with COVID-19 meant that there was no need to be vaccinated. It has been shown that some patients who contract COVID-19 infection may not produce long-lasting antibodies against the virus, making them vulnerable to reinfection, ^40^ which makes vaccination necessary, even with a previous infection. Another false belief was that the COVID-19 vaccine could lead to infertility; in our survey, 11.6% of respondents either agreed or strongly agreed with this rumor. Another a recent study proved that such claims were incorrect. ^41^

We investigated the relationship regarding acceptance of misconceptions with predictors including sex, age, educational level, occupation, and sources of information. We found sex to be a strong positive predictor of acceptance of misconceptions (p=0<001). Our results showed that, on average, women had greater acceptance of misconceptions than men. This result has been confirmed in surveys conducted among Jordanian students where male students had a greater intent to be vaccinated. This may be attributed to men perceiving the disease as being more dangerous than women. ^42^ This finding was also reported in a study in the United States, in which women were less accepting of COVID-19 vaccination than men. ^43^ A greater tendency to accept vaccination among men than women might be partly explained by a greater non-acceptance of rumors than women. Another survey conducted in Saudi Arabia supported this result, showing that men have greater compliance with vaccination than women, although the difference was not significant. ^44^

All professions in this survey were found to be negative predictors of the acceptance of misconceptions, but the associations were not significant. Pensioners were more likely to disagree with misconceptions compared with other professions, but the association was not significant. However, contradictory results were reported in a survey conducted in Qatar; retired participants were reported to be more hesitant to be vaccinated. ^45^ In our study, pensioners, followed by students, were more likely to disagree with misconceptions compared with other occupations, although the association regarding students was also not significant. This result is supported by a study where Jordanian students in health school accepted the COVID-19 vaccines more than did other groups. ^46^

The age group 18–30 years showed the greatest association with negative prediction of accepting misconceptions, but the association was not significant. This contradicts another survey where participants who believed in conspiracy theories were slightly younger than those who did not believe conspiracy theories. ^47^ Our findings are supported by those of a study conducted in the United Kingdom, in which vaccine-hesitant individuals were more likely to be under age 65 years. ^5^ Another survey conducted by Tamam El-Elimat and colleagues showed that younger individuals tended to accept vaccination more than other age groups. ^48^

Education level is an essential predictor of the acceptance of misconceptions. Our results indicated that the educational levels secondary school and university or above were associated with decreased acceptance of rumors, and the association was significant at the secondary school level (p=0.001). Our survey also showed that having less than a secondary school education is a positive predictor of accepting misinformation; however, the association was not significant. Our finding is supported by a study in the United States, which concluded that low literacy levels were associated with reluctance to be immunized. ^49^ However, our findings were in contrast to those of a study showing that well-educated people have lower rates of accepting vaccination. ^50^ However, such rejection can be attributed to other factors because misconceptions and rumors are not the only factors affecting vaccination hesitancy.

Most of our respondents (60.5%) reported using social media platforms like Facebook, Whatsapp, and Twitter as their main source of information in relation to COVID-19. Another survey reported that only 16% of their study population used Facebook as a source of information, and 16% obtained their information from traditional media sources. ^51^ A strong significant association was found in our survey between the source of information and acceptance of misconceptions whereas other studies have indicated that 30%–60% of the information on social media platforms has an anti-vaccine tendency and over 50% of the information on vaccine-related websites is false or inaccurate. ^22^ This may contribute to the spread of false information regarding COVID-19 vaccines among the population.

It must be noted that this study has some limitations as it aimed mainly to assess the distribution of acceptance of misconceptions among participants and the factors that can increase or decrease this acceptance, but it didn’t measure the willingness to be vaccinated among the same respondents, more surveys are needed to assess the correlation between distribution of misconceptions and willingness to be vaccinated.

## Conclusions

The results of our study showed that the most accepted misconception among respondents in Saudi Arabia was that COVID-19 vaccines have serious side effects like causing allergy, followed by the rumor that COVID-19 vaccines are unsafe. This should raise a red flag for policymakers to address the spread of misinformation by increasing awareness among the public using the most popular platforms, namely, social media, and implementing additional measures aimed at reducing the spread of COVID-19 vaccine-related misinformation and rumors.

## Data availability

### Underlying data

Dryad: Misconceptions about COVID-19 vaccine among adults in Saudi Arabia and their associated factor, https://doi.org/10.5061/dryad.2jm63xsr9. ^52^

This project contains the following underlying data:

- Misconceptions.csv

Data are available under the terms of the Creative Commons Zero “No rights reserved” data waiver (CC0 1.0 Public domain dedication).

### Extended data

Zenodo: Misconceptions about COVID-19 vaccine among adults in Saudi Arabia and their associated factor, https://doi.org/10.5281/zenodo.6385577. ^53^

This project contains the following extended data:

- Figure_files.doc
- Table_file.doc

Data are available under the terms of the Creative Commons Attribution 4.0 International license (CC-BY 4.0).
