# Supplementary material for: Misconceptions about COVID-19 vaccine among adults in Saudi Arabia and their associated factors: A cross-sectional study conducted in 2021
Source: F1000Res. 2023 Dec 27;11:561. Originally published 2022 May 23. [Version 2] doi: 10.12688/f1000research.110270.2 (PMC10933568; doi:10.12688/f1000research.110270.2)
Supplement: Peer review history

# Reviewer report: Reviewer response for version 2

**DOI:** 10.5256/f1000research.160473.r233761

**Published:** 2024-03-12

**Authors:** Muhammad Chutiyami

**Affiliations:** 1 School of Nursing and Midwifery, University of Technology Sydney, Sydney, Australia

Thank you for the opportunity to review this manuscript again. My earlier comments have been addressed reasonably.

 The only minor thing that needs to be added to the final proof is to indicate the total number of respondents in the abstract, otherwise, the percentages indicated in the abstract result would not make full sense.

 The paper is now well revised without the need for further review from my end.

Is the work clearly and accurately presented and does it cite the current literature?

Yes

If applicable, is the statistical analysis and its interpretation appropriate?

Yes

Are all the source data underlying the results available to ensure full reproducibility?

Yes

Is the study design appropriate and is the work technically sound?

Yes

Are the conclusions drawn adequately supported by the results?

Yes

Are sufficient details of methods and analysis provided to allow replication by others?

Yes

Reviewer Expertise:

Infectious diseases, immunization, community health nursing

I confirm that I have read this submission and believe that I have an appropriate level of expertise to confirm that it is of an acceptable scientific standard.

# Reviewer report: Reviewer response for version 1

**DOI:** 10.5256/f1000research.121856.r199319

**Published:** 2023-08-30

**Authors:** Muhammad Chutiyami

**Affiliations:** 1 School of Nursing and Midwifery, University of Technology Sydney, Sydney, Australia

Thank you for the opportunity to review the manuscript. Below are some minor comments to improve the manuscript;

 There is a need for a clearer justification of the 11 items to assess misconception. The current information under survey items; ‘second section addressed 11 misconceptions derived from the literature, the community, social media, and websites’ is verge. At a minimum, you can cite some of the literature sources. You could also acknowledge the lack of validation of the questionnaire as a limitation. Also, acknowledge the limitation that the data used was collected in 2021, and that it is possible those misconceptions have changed currently.

 Please indicate the source of the sample size formula.

 In your logistic regression analysis (Table 4), it is important to add the reference category under each variable e.g. male, 61-70 years, etc. This will ease the interpretation of the table.

 In the discussion (5 ^th^ paragraph), it is important to reflect more on the vaccine's actual severe/long-term side effects, which may influence those misconceptions, rather than accepting all severe side-effects as misconceptions.

Is the work clearly and accurately presented and does it cite the current literature?

Yes

If applicable, is the statistical analysis and its interpretation appropriate?

Yes

Are all the source data underlying the results available to ensure full reproducibility?

Yes

Is the study design appropriate and is the work technically sound?

Yes

Are the conclusions drawn adequately supported by the results?

Yes

Are sufficient details of methods and analysis provided to allow replication by others?

Yes

Reviewer Expertise:

Infectious diseases, immunization, community health nursing

I confirm that I have read this submission and believe that I have an appropriate level of expertise to confirm that it is of an acceptable scientific standard, however I have significant reservations, as outlined above.

# Reviewer report: Reviewer response for version 1

**DOI:** 10.5256/f1000research.121856.r182219

**Published:** 2023-07-18

**Authors:** Jessica Carter

**Affiliations:** 1 Griffith University, Nathan, Queensland, Australia

Thank you for the opportunity to review this interesting and important study. Overall, this research is sound and offers a clear outline of methods, results and the implications of these. 

 Areas of suggestion are minor but would improve readability and replicability:

- Introduction: much of the detail here, particularly information on the vaccine development, could be reduced. The data given from studies outside of Saudi Arabia could also be shortened and/or made clearer why these are applicable to your study's population. The inclusion of definitions of vaccine hesitancy and refusal, and "misconception", would be useful. 
- Methods: rationale for the choice of 11 statements would be valuable. This may be relevant to include in Introduction as supporting content around dis/misinformation and vaccine hesitancy. Perhaps also worth explaining the reason not to measure COVID-19 vaccine acceptance/willingness to be.
- Methods: this section notes a 4-point scale but Abstract notes a 5-point. Please ensure all references to the scale are consistent and correct. 
- General: language is wordy at points, particularly in the Abstract and Introduction. Consider revising these sections for greater clarity and conciseness.

Is the work clearly and accurately presented and does it cite the current literature?

Yes

If applicable, is the statistical analysis and its interpretation appropriate?

Yes

Are all the source data underlying the results available to ensure full reproducibility?

No source data required

Is the study design appropriate and is the work technically sound?

Yes

Are the conclusions drawn adequately supported by the results?

Yes

Are sufficient details of methods and analysis provided to allow replication by others?

Yes

Reviewer Expertise:

Public Health; Social Determinants of Health; Behaviour Change and Health Promotion; Prevention Science; Vaccine Hesitancy.

I confirm that I have read this submission and believe that I have an appropriate level of expertise to confirm that it is of an acceptable scientific standard.
